# Introducing a healthcare-assistive robot in primary care: a preliminary questionnaire survey

**DOI:** 10.3389/frobt.2023.1123153

**Published:** 2023-05-11

**Authors:** N. C. Tan, Y. Yusoff, D. Koot, Q. C. Lau, H. Lim, T. F. Hui, H. Y. Cher, P. Y. A. Tan, Y. L. E. Koh

**Affiliations:** ^1^ SingHealth Polyclinics, Singapore, Singapore; ^2^ School of Life Sciences & Chemical Technology, Ngee Ann Polytechnic, Singapore, Singapore; ^3^ School of Health Sciences, Ngee Ann Polytechnic, Singapore, Singapore; ^4^ School of Engineering, Ngee Ann Polytechnic, Singapore, Singapore; ^5^ Yong Loo Lin School of Medicine, National University of Singapore, Singapore, Singapore

**Keywords:** healthcare-assistive robot, primary care, patient, healthcare worker, infection control

## Abstract

A Healthcare-assistive Infection-control RObot (HIRO) is a healthcare-assistive robot that is deployed in an outpatient primary care clinic to sanitise the premises, monitor people in its proximity for their temperature and donning of masks, and usher them to service points. This study aimed to determine the acceptability, perceptions of safety, and concerns among the patients, visitors, and polyclinic healthcare workers (HCWs) regarding the HIRO. A cross-sectional questionnaire survey was conducted from March to April 2022 when the HIRO was at Tampines Polyclinic in eastern Singapore. A total of 170 multidisciplinary HCWs serve approximately 1,000 patients and visitors daily at this polyclinic. The sample size of 385 was computed using a proportion of 0.5, 5% precision, and 95% confidence interval. Research assistants administered an e-survey to gather demographic data and feedback from 300 patients/visitors and 85 HCWs on their perceptions of the HIRO using Likert scales. The participants watched a video on the HIRO’s functionalities and were given the opportunity to directly interact with it. Descriptive statistics was performed and figures were presented in frequencies and percentages. The majority of the participants viewed the HIRO’s functionalities favourably: sanitising (96.7%/91.2%); checking proper mask donning (97%/89.4%); temperature monitoring (97%/91.7%); ushering (91.7%/81.1%); perceived user friendliness (93%/88.3%), and improvement in the clinic experience (96%/94.2%). A minority of the participants perceived harm from the HIRO’s liquid disinfectant (29.6%/31.5%) and that its voice-annotated instructions may be upsetting (14%/24.8%). Most of the participants accepted the HIRO’s deployment at the polyclinic and perceived it to be safe. The HIRO used ultraviolet irradiation for sanitisation during after-clinic hours instead of disinfectants due to the perceived harm.

## Introduction

The coronavirus (COVID-19) pandemic has heightened the awareness of infection control globally. Infection control measures are introduced in local communities to curb the spread of COVID-19, such as mandatory mask-wearing, crowd control and segregation in the community, mass vaccinations, and regular sanitisation of public and healthcare facilities (Shbaklo et al., 2021). Despite the introduction of these measures, over 6.3 million people have died from infections, of which 115,000 of them are HCWs (World Health Organization, 2021). Many more HCWs have been infected, resulting in a depletion of the workforce at the frontline. Aside from COVID-19, endemic pathogens continue to thrive in the environment, which requires regular anti-infective measures to keep them under control. The emergence of novel pathogens raises the demand of healthcare services in the community. Furthermore, reliance on human resource to implement infection control measures can be difficult to sustain amidst a shortage of healthcare manpower.

Traditionally, advances in robotic technology have been made mainly in the manufacturing industry due to the need for collaborative robots. However, this is not the case in service sectors, especially in the healthcare sector (Holland et al., 2021). Prior to the pandemic, the World Health Organization (WHO) had advocated the deployment of assistive robots in healthcare in 2018: “The impact of assistive technology goes far beyond the benefits of health and well-being to individual users and their families. It also has socioeconomic benefits, by reducing direct health and welfare costs, enabling a productive labour force, and stimulating economic growth” (World Health Organization, 2017). In 2019, the market for professional service robots expanded by 32% (International Federation of Robotics, 2020). The rapidly evolving robotic technology provides a potential solution to alleviate the burden on overworked healthcare workers, such as those in the community (Kritikos, 2020).

The spread of COVID-19 was previously prevalent in Singapore due to the emergence of highly infectious XBB Omicron sub-variants (Today, 2021). Public primary care clinics (polyclinics) are the first line of healthcare contact for the majority of the local multi-ethnic Asian population. Each of the 23 polyclinics across three regional health systems on the island state serves approximately 500 to over 1,100 patients daily during office hours. High patient loads are a potential health hazard for cross-infection within the polyclinic despite the segregation of suspected patients for the management of their acute symptoms. More frequent cleaning of the polyclinic premises is necessary to prevent such cross-infections, but its implementation is a challenge due to manpower constraints and hindrance from crowds. Tech-enabled solutions, such as the deployment of healthcare-assistive robots, may potentially address the current barriers towards safer healthcare premises for patients and HCWs.

SingHealth Polyclinics (SHP) is a public primary care provider and academic family medicine institution comprising a network of nine polyclinics (primary care clinics) in the eastern region of Singapore. It has collaborated with a robotics training centre in a centre of higher learning (Ngee Ann Polytechnic) to develop a multi-functional healthcare-assistive infection-control robot (HIRO). The mobile HIRO is capable of sanitising the surroundings using ultraviolet irradiation, identifying humans with improper mask donning, screening them for fever, and providing information on the location of service points in a polyclinic. It was deployed for a feasibility pilot trial in a large and busy polyclinic in SHP to assess the human–robot interaction (HRI) before scaling up across the rest of the polyclinics.

HRI is a field of knowledge dedicated to understanding, designing, and evaluating robotic systems for use by or with humans (Huang, 2016). Robots have the possibility to interact with people and the environment based on their programmed functions. However, limited evidence has revealed that people do not seem to entirely trust and accept robots (Pinney et al., 2022), and prefer to have people instead of robots interact with them in the healthcare setting. Multiple studies have investigated elderly patients’ perceptions to robotic symptoms at home or in the hospital setting (Mois and Beer, 2020; Cortellessa et al., 2021). However, similar studies relating to robot deployment in outpatient primary care settings with patients and HCWs of various ages are limited. The level of acceptability, safety perception, and concerns of the public and HCWs are postulated to be the major determinants of the successful deployment of robots to fulfil their functions in healthcare settings.

This study aimed to determine the acceptability, perceptions of safety, and concerns among patients, their accompanying persons, and onsite primary HCWs regarding the HIRO when it was deployed for a trial run at a polyclinic in Singapore.

## Methods

### Survey site

A cross-sectional questionnaire survey was conducted at Tampines Polyclinic, a primary care clinic that provides healthcare to the residents in an estate in eastern Singapore. This polyclinic serves approximately 1,000 patients and visitors daily and has a staff strength of 170 HCWs.

#### HIRO

**FIGURE 1:**
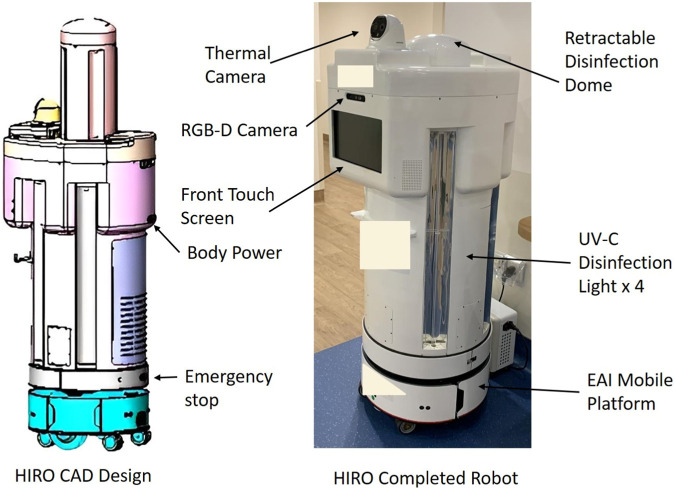
Structure of the HIRO.

The HIRO consists of three modules: a mobile platform module, HIRO body module, and disinfection dome module. Figure 1 shows the structure of the HIRO.

1) Mobile platform module

The Enterprise Application Integration mobile platform has a built-in SLAM navigation package with two high-precision G42D LiDAR and an IMU. It is powered by two 24 V DC-geared motors and supported by four casters with suspension. The cruise speed with a payload is approximately 0.5 m/s.

2) HIRO body module

The HIRO body module has been installed with four 75 W UV-C lights for horizontal disinfection. The thermal camera monitors human temperature. The RGB-D camera observes any human in front of the robot and executes mask detection if anyone is observed. The front screen provides the user interface (UI) for two interactive applications: 1) for users looking for a facility/consultation room in the polyclinic and 2) for HCWs to configure the route path and schedule for UV-C light disinfection.

3) Disinfection dome

The disinfection dome can retract into the HIRO’s body for safety reasons, and before disinfection starts, the dome rises from the HIRO’s body. It was originally designed to sanitise the surroundings with disinfectant liquid, and the method has since been changed after feedback. It is equipped with four 28 W UV-C lights. It sanitises the upper horizontal space and ceiling. The disinfection efficacy is 99.9% for a duration of 3 min at a maximum distance of 1 m.

4) Safety performance evaluation

The HIRO underwent a safety performance evaluation in the simulation laboratory at the School of Health Sciences in Ngee Ann Polytechnic, which included tests for disinfection and object detection. The HIRO applies distance-based speed control algorithms for navigation. It is equipped with two front obstacle avoidance sensors at a height of 13.4 cm. The obstacle avoidance sensors detect objects on the floor. The speed control algorithm will slow down the HIRO when it detects an object at a distance of 30 cm and stops at a distance of 15 cm.

#### Period of study

Participants were recruited from March to April 2022. During the period of study, the HIRO was deployed during office hours (8 a.m. to 5 p.m.) at the polyclinic to carry out all of its tasks, except sanitisation. A schedule was drawn up for the HIRO to be stationed at the entrance area of the polyclinic for human temperature monitoring, detecting improper mask wear, and location finding. When the HIRO was deployed during office hours, HCWs, patients, and visitors would have the opportunity to interact with the robot by using its touchscreen for way finding and by standing in front of the HIRO for temperature detection. For safety reasons, interactions between participants and the HIRO for detection of mask wearing was not possible as it was mandatory for all persons in the clinic to be masked. The sanitisation task using ultraviolet irradiation was executed after office hours. The HIRO’s interactions with people were extensively reviewed and tested by the engineers before its deployment in the study.

#### Study population computation

As there was no prior literature for reference to compute the study size, a proportion of 0.5 was used to attain the maximum variability of the outcomes. With a precision of 5% and a confidence interval at 95%, the sample size using the proportion of 0.5 was estimated to be 385.

#### Eligibility criteria

The target participants included the public and HCWs who were at the designated patient waiting area in the vicinity of the HIRO during its deployment at the polyclinic. Participants who could not converse or comprehend English were excluded from the study. The public comprised patients receiving care at the polyclinic, their accompanying persons, and visitors. HCWs included primary care doctors, nurses, pharmacy staff, allied health professionals, and administrative and support staff.

#### Study procedure

Two research assistants were trained by the investigators to approach potential participants of varying demographic profiles at the study site to administer the questionnaire. The research assistants approached target participants, provided relevant information about the study, and obtained their verbal consent before executing the study.

The participants were shown a short 3-min video (https://youtu.be/147NZ0Vi6ds) on a tablet to introduce and showcase the functionalities of the HIRO. After participants had viewed the video, they were given the option to voluntarily interact with the HIRO or to proceed with answering the questionnaire survey without interacting with the HIRO. The research assistants administered the digital questionnaire using a tablet leveraging on an online survey platform (https://form.gov.sg). No monetary incentive was offered to the participants.

#### Questionnaire

The survey consisted of four sections, comprising closed- and open-ended questions. The participants self-administered the questionnaire or were assisted by the research assistants to gather their personal views on the functionalities of the robot on a four-point Likert scale (strongly agree, agree, disagree, and strongly disagree). Data were also collected on their safety concerns regarding the deployment of the HIRO at the study site.

#### Data management and audit

Survey responses were extracted, coded, and entered into a database by trained research personnel. The data were stored in a password-protected computer accessible only by the study team members.

### Statistical analyses

Descriptive statistics were performed, and figures were presented in frequencies and percentages. The responses to questions, such as “strongly agree” and “agree”, were assigned to one group and those of “strongly disagree” and “disagree” to another group. A chi-square test was used to assess the association between the demographical profiles of the participants and their perceptions on the robot. A *p*-value of less than 0.05 was considered to be statistically significant. IBM SPSS 27.0 was used to compute the analysis.

## Results

**FIGURE 2:**
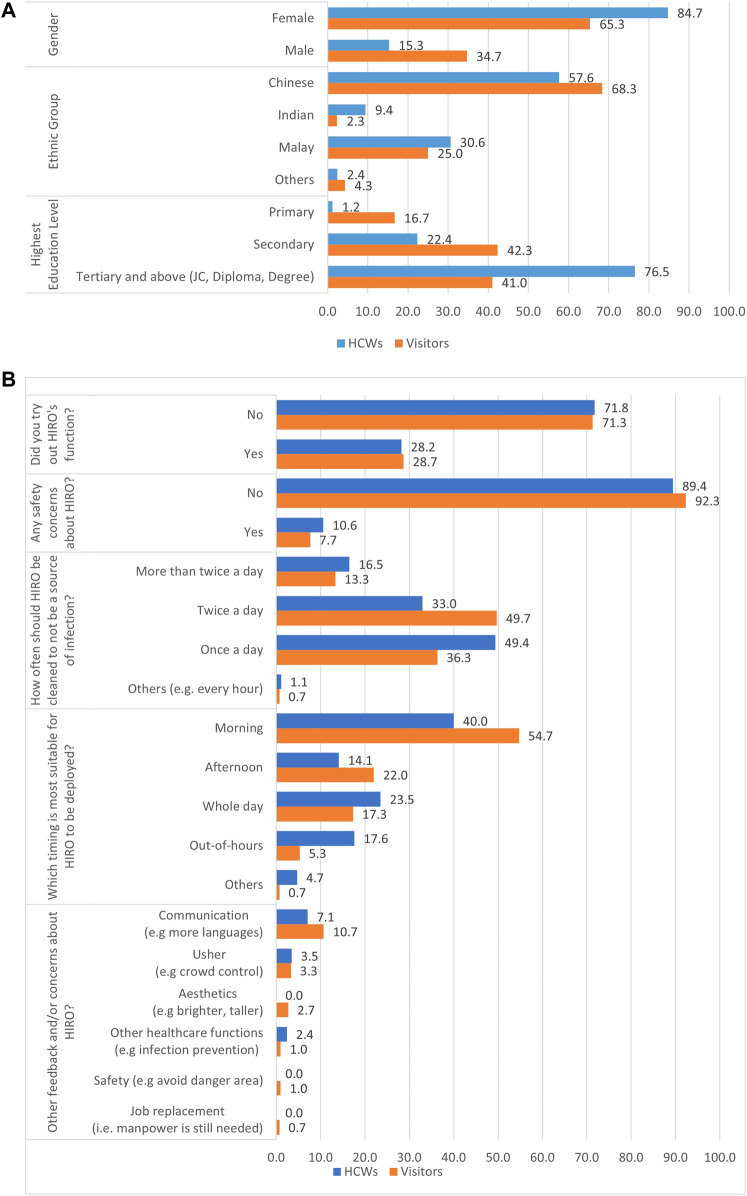
**(A)** Demographic profiles of HCWs and visitors and **(B)** HCWs’ and visitors’ views on the HIRO.

Figure 2A shows the demographic characteristics of the subjects. Of the 385 respondents, 85 (22.1%) were the polyclinic's HCWs, while the rest were patients and visitors, with 69.6% being women, 66.0% of Chinese ethnicity, and 48.8% having post-secondary or tertiary education.

Figure 2B shows that the majority of the participants (89.4% of HCWs and 92.3% of patients/visitors) had no safety concerns regarding the robot, but only 28.6% tested its function directly. Most of them (40.0% of HCWs and 54.7% of patients/visitors) opined that the HIRO should be deployed in the mornings.

**FIGURE 3:**
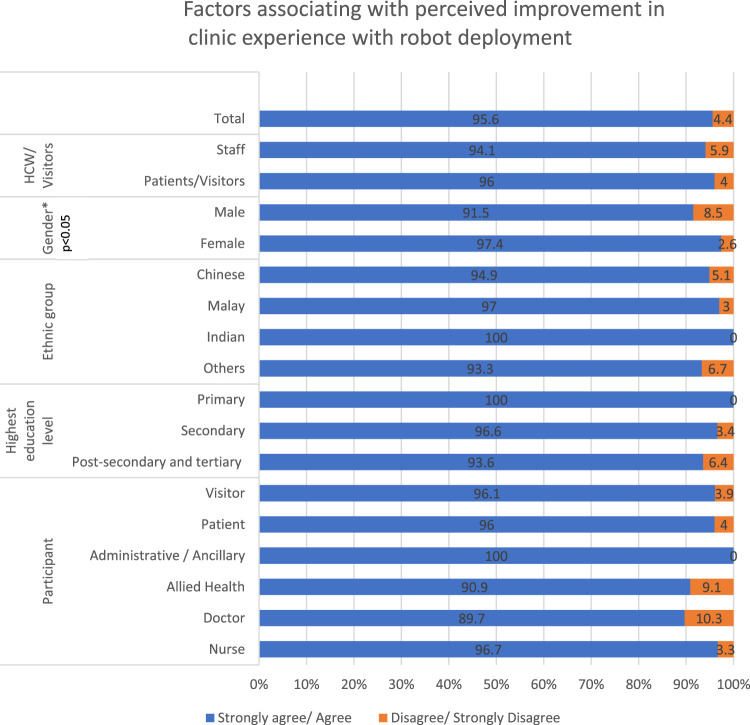
Factors associated with the perceived improvement in the clinic experience with robot deployment.

The results from Figure 3 show that women were more likely to perceive that the HIRO could help in improving the polyclinic experience when compared with men. No other significant demographic difference was noted between the groups.

### Participants’ views on the functionalities and performance of the HIRO

**FIGURE 4:**
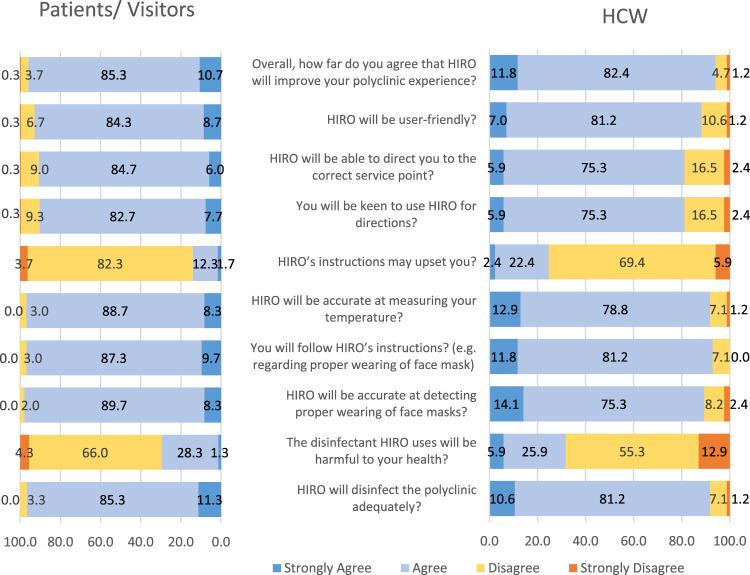
Views on the functionalities of the HIRO from patients/visitors and HCWs.

Figure 4 shows that approximately 90.7% of the patients/visitors, when compared to 81.2% of the HCWs, agreed that the HIRO could direct them to the correct service points, and 97.0% of the patients/visitors and 91.7% of the HCWs agreed that the HIRO could assess their body temperature. Almost all (98.0%) of the patients/visitors, when compared to 89.4% of the HCWs, agreed that the HIRO would be capable of identifying people who had improper mask donning. More patients/visitors when compared to HCWs (96.7% vs. 91.8%) agreed that the HIRO could disinfect the polyclinic adequately; overall, a majority of the participants (96.0% of the patients/visitors and 94.2% of the HCWs) agreed that the HIRO could improve their clinic experience.

### Use of HIRO by respondents

Figure 4 shows a larger proportion of the patients/visitors (90.4%), when compared to the HCWs (81.2%), agreed that they would be keen to use the HIRO for directions; 93.0% of the patients/visitors and 88.2% of the HCWs agreed that the HIRO would be user-friendly. A total of 97.0% of the patients/visitors and 93% of the HCWs agreed that they would follow the HIRO’s instructions regarding the proper donning of face masks.

### Respondents’ concerns regarding HIRO

Approximately 30% of the respondents agreed that the disinfectant that the HIRO used would be harmful to their health. A total of 7.7% of the patients/visitors and 10.6% of the HCWs had safety concerns regarding the HIRO. Out of these groups of participants who had safety concerns, 5 in 9 HCWs and 16 in 23 patients/visitors agreed that they might trip over the HIRO, and a majority of them (87% of the visitors and 78% of the HCWs) agreed that the HIRO may be damaged by visitors. Out of all 385 participants, a small proportion of 16.4% agreed that the HIRO’s instructions may upset them.

### Perceived functionalities to add to the healthcare-assistive robot in an ambulatory primary care setting

Functions that the participants suggested that they would want the HIRO to perform included greeting patients, helping crowd control, announcement of information, announcement of emergency alerts, and carrying out nasal or throat swabs to detect COVID-19. Other functionalities proposed by participants included automatically raising alarm or other signals to indicate the robot’s intended path to avoid collision and injuries, the ability to communicate in different local languages, and offering reception services.

### Perceived safety and other concerns

Respondents suggested aesthetic changes such as to paint the robot in bright colours for visual cues or have a taller stature. One concern was the risk of job replacement of HCWs when such robots are deployed on a large scale in primary care.

## Discussion

Robots are increasingly deployed in healthcare settings to support HCWs and to enhance patient care. The implementation of new technologies in an existing healthcare environment may result in cultural changes, which may potentially impact social interactions and result in positive and negative emotions for HCWs and their patients. While technological acceptance, such as of safety and usefulness, is often reported in the literature, the issue of emotional management receives less attention. Nonetheless, effectively managing the emotional status of these stakeholders is the key to the wider adoption of the technology by institution leaderships and policymakers. It will also facilitate the adaptation of HCWs and patients to the presence of healthcare-assistive robots in their proximity. In 2015, Hersh (2015) advocated a need for deeper understanding of ambivalent user acceptance. In a 2019 systematic review, Krick et al. (2019) recommended three core outcome dimensions relating to digital technologies for all participants involved in the care sectors by evaluating the acceptance, effectiveness, and efficiency of new technologies.

Aligned to the recommendation, this survey aimed to assess the acceptance of the HIRO by primary HCWs and the patients whom they served. The results show a high level of acceptance and perceived effectiveness of the HIRO in delivering its functions by at least 9 out of 10 participants from both the groups (Figure 4). Overall, the majority of the participants felt that the functionalities delivered by the robot would enhance their experience during their visit to the polyclinic. Similarly, a survey of 166 Chinese respondents by Jiang and Cheng (2021) had also revealed positive attitudes towards “anti-pandemic robots” that aimed to “reduce the burden of medical care and virus transmission.” The results inform the robotic industry about the feasibility of deploying more service robots in primary care and community settings with suggestions to improve its design and functionalities. The HIRO can also be deployed in tertiary healthcare settings to relieve the limited precious manpower from manual laborious and repetitive tasks.

Nevertheless, approximately 1 in 20 participants were less receptive to the robot's functionalities. This could be related to their prior knowledge of, interests in, and experiences with robotic systems, or possibly the time taken to introduce the robot to the participants. HCWs’ prior experience and length of exposure to the robot could result in differences in results between healthcare professionals and patients/visitors. More information could be disseminated to raise public awareness of the HIRO using various media, from printed matter to videos that can be broadcasted in polyclinics. Deliberate, hands-on opportunities to familiarise targeted users with the HIRO will be created before its future implementation to address the observation that only 28.6% of the participants had tested its functions during the survey.

While the majority of the participants (95.6%) had perceived that the HIRO would be capable of disinfecting the polyclinic premise, almost a third of the participants (30.1%) reported their concern regarding the potential harm from disinfectants. The plan is to deploy the HIRO for this role outside clinic hours to eliminate any possible UV-radiation injury, which is possible with the automation of the robot and its autonomous navigation capability. However, the majority of the participants felt that the robot should be operational during clinic hours, suggesting a knowledge gap that can be addressed with relevant information on the safe modus operandi of the HIRO.

Most participants reflected that they would follow the robot’s instructions to don a mask if alerted to the lapse, but 16.4% of them alluded to the potential of feeling upset from its broadcasted reminder. This dismay could arise from personal embarrassment or perceived intimidation, possibly due to the presence of the public and onlookers in the polyclinic. However, the recorded message from the HIRO is not directed at any specific person but consists of a general reminder when it identifies a person without the proper donning of a mask in its proximity. According to Hopko et al. (2022), “the degree of successful human-robot collaboration is dependent on the joint consideration of robot factors and human factors”, which is reflected in this result. Beyond the perception reported in this survey, further studies are required to observe the range of human–robot interactions, which should include reactions to verbal reminders from the HIRO when it is implemented routinely in polyclinics.

Safety is of prime concern to the participants. Safety underpins healthcare-assistive robot applications and human–robot coexistence. A total of 87.5% of participants cited their concern regarding injuries resulting from collision with the moving robot. Prior to deployment, the HIRO’s safety performance was assessed at Ngee Ann Polytechnic. Future actions that are proposed to enhance the HIRO’s safety performance will include implementing downward staircase detection using AI and deep learning algorithms, adding a visible warning light to alert it to its surroundings when the HIRO is in use, and ensuring that the robot can navigate elevators if further funding can be secured.

Several participants have articulated their concern about job replacement by the robot through qualitative feedback, which was not covered by the questionnaire survey. Such concerns were also raised by respondents in the survey that was carried out by Jiang and Cheng (2021). Healthcare-assistive robots are perceived to take over the jobs of humans in healthcare settings as they are not at risk of infection and hence, do not require face masks or social distancing. These robots will not be embroiled in labour conflicts for better personal welfare and will assume an advantageous role to replace the unskilled labour force. Nonetheless, with a highly educated population in Singapore, unskilled labour has to be imported from neighbouring countries, which can become unsustainable in the near future as regional economies grow and require expansion of labour forces to operationalise their industries. The public have to be reassured that these robots aim to free the polyclinic HCWs from manual mundane tasks such as disinfection and allow them to perform patient-fronting healthcare services. Nevertheless, the participants have indicated that the need for HCWs to clean and sanitise the robot twice daily (Figure 2B) to prevent it from becoming a source of infection requires human labour. The optimal frequency of cleansing will be evaluated in future studies.

Robots in healthcare currently have been grouped in various ways as have been shown by multiple studies (Kyrarini et al., 2021; Vallès-Peris et al., 2021). When compared to other commercially available robots, the HIRO is a novel hybrid robot that is healthcare assistive and can interact with the end user because of its multiple functionalities. For example, the UVD (Astrid et al., 2021), HERO21 (ICA Group, 2023), and LightStrike (Nikkei Asia, 2023) are mobile disinfectant robots. The ARIS-K2 YOUIBOT (Servo Dynamics Pte Ltd, 2023) is an autonomous mobile robot that eradicates viruses with UV light and is equipped with infrared temperature checking technology. The HIRO has the capability of identifying improper mask wearing as well as ushering capabilities. Among the four commercially available robots, only a study on the UVD robot has been published (Astrid et al., 2021), which focused on the efficacy of its disinfection function. Hence, the scientific literature on commercially available robots seems sparse, especially that relating to the robot–human interaction and the perception of safety and acceptability of end users. Our study aims to fill this gap and to highlight the variable perceptions of people encountering and interacting with a robot in primary care.

The qualitative feedback from the study participants underpins future improvements to the robot. Future rollout plans include cost saving analyses when the robot is deployed to more primary care sites. However, scaling up its implementation requires funding and investment to create more robots. Replicating the study in other clinics may uncover variations in the results and potentially enhance the generalisability. The results will provide an effect size to compute the size of the study population, such that an adequately powered study can be conducted to determine the outcomes stratified by the demographic factors. The slightly lower ratings towards the HIRO found among the HCWs allude to the potential effect of prior knowledge and experience with robots on their responses to the survey. The results provide an impetus to consider a qualitative research study to deep dive into the robot–healthcare professional interaction in the near future.

The study has its limitations. The case-encounter mode of recruitment constitutes a potential source of bias. The study did not include HCWs, patients, or visitors who could not comprehend or speak English. Participants were also given the choice to test the HIRO’s functions on a voluntary basis, but only 28.6% of them tested the HIRO’s functions directly, which could have had an impact on their perceptions. Separating the outcomes of the two groups to evaluate if the physical interaction with the robot changes the feeling/reactions of the participants would be useful. These limitations may restrict the generalisability of the results. Participants’ prior knowledge and experience with robots were not surveyed, and this may have implications on the results of the survey. The number of HCW participants was disproportionate to that of patients and visitors due to the smaller staff strength at the study site compared to the large patient pools that the polyclinic serves. Nonetheless, constructive feedback and acceptance from both groups of participants were essential for the HIRO to be deployed at the primary care clinic.

## Conclusion

The majority of patients/visitors and HCWs were receptive to the HIRO’s deployment at the polyclinic and favourably perceived its functionalities and safety. Their feedback contributed to a switch from disinfectant to ultraviolet irradiation as the mode of disinfection.

## Data Availability

The raw data supporting the conclusion of this article will be made available by the authors, without undue reservation.
