# Corrigendum: Introducing a healthcare-assistive robot in primary care: a preliminary questionnaire survey

**DOI:** 10.3389/frobt.2023.1224492

**Published:** 2023-05-30

**Authors:** N. C. Tan, Y. Yusoff, D. Koot, Q. C. Lau, H. Lim, T. F. Hui, H. Y. Cher, P. Y. A. Tan, Y. L. E. Koh

**Affiliations:** ^1^ SingHealth Polyclinics, Singapore, Singapore; ^2^ School of Life Sciences & Chemical Technology, Ngee Ann Polytechnic, Singapore, Singapore; ^3^ School of Health Sciences, Ngee Ann Polytechnic, Singapore, Singapore; ^4^ School of Engineering, Ngee Ann Polytechnic, Singapore, Singapore; ^5^ Yong Loo Lin School of Medicine, National University of Singapore, Singapore, Singapore

**Keywords:** healthcare-assistive robot, primary care, patient, healthcare worker, infection control

Incorrect Funding

In the published article, the **Funding** statement was incomplete as funding for the publication cost from another grant was left out. The original text is “Robot development was supported by the Decentralised Gap Fund (DGF 20GAP006) awarded to Ngee Ann Polytechnic from the Singapore Ministry of Education. The survey was conducted pro-bono by the primary healthcare investigators from SingHealth Polyclinics.” The correct Funding statement appears below.

The correct **Funding** statement is “Robot development was supported by the Decentralised Gap Fund (DGF 20GAP006) awarded to Ngee Ann Polytechnic from the Singapore Ministry of Education. The survey was conducted pro-bono by the primary healthcare investigators from SingHealth Polyclinics. The publication cost was supported by Singhealth Polyclinics Centre Grant CG21APR3006 (NMRC/CG3/001/2022-SHP).

The authors apologize for this error and state that this does not change the scientific conclusions of the article in any way. The original article has been updated.

